# Eating attitudes: The extent and risks of disordered eating among amateur athletes from various sports in Gauteng, South Africa

**DOI:** 10.4102/sajpsychiatry.v24i0.1179

**Published:** 2018-08-29

**Authors:** Rudolph L. van Niekerk, Melissa Card

**Affiliations:** 1 Department of Human Movement Sciences, University of Fort Hare, South Africa; 2 Department of Psychology, University of Johannesburg, South Africa

## Abstract

**Background:**

Preoccupation around eating and disordered eating of professional athletes has extensively been discussed in the literature. However, the extent of disordered eating behaviours at the non-professional or amateur club level in South African sport has not received the same amount of attention.

**Objectives:**

This study attempted to determine the extent of disordered eating behaviours among amateur athletes to identify the athletes at risk of developing an eating disorder. Group differences and predictive factors were explored to determine factors associated with disordered eating behaviours among amateur sporting athletes.

**Methods:**

A purposive sample of athletes (*n* = 278) with a mean age of 27 ± 11.30 years, from various sports clubs in Central Gauteng (in and around Johannesburg), were asked to complete the Eating Attitudes Test-26 and Sport Competition Anxiety Test. Data analysis included descriptive statistics, independent samples *t*-tests, analysis of variance (ANOVA) and logistic regression.

**Results:**

The results indicated that 14.7% of athletes were at risk of developing an eating disorder, while some engaged in excessive weight control behaviour which put them at risk. Gender and weight control strategies were important indicators associated with the risk of developing an eating disorder. The athletes’ gender, level of participation and body mass index (BMI) were important predictors of the risk to develop an eating disorder.

**Conclusion:**

Indicators of eating disorder risk among club-level amateur athletes are gender, binge eating, vomiting and using laxatives to control weight. These behaviours predominately found in female athletes seem to put them at a greater risk of developing an eating disorder.

## Introduction

Eating attitudes and disordered eating behaviours among athletes is an area of research that needs much attention. This research attempts to address this problem by assessing disordered eating behaviour among athletes from various amateur sports. The general meaning of the term ‘athlete’ will be used in this article to refer to a sports participant in any of the sports included in the study. Within South Africa, there is no real focus on how athletes engage with eating and food in general. However, there is much focus on the prevalence of eating disorders in athletes, which seems to be similar to international research, and leads to the conceptualisation of the female athlete triad^1^ – that is, an interrelationship between disordered eating behaviours, amenorrhea and low bone density – which attests to the significant health risks for women participating in sport and places them at high risk of developing an eating disorder. One of the factors in this triad is disordered eating behaviours, which is seen as an inclination towards rapid weight loss and abnormal eating behaviours that are subclinical, as they do not meet the criteria for any diagnosable eating disorder. Over time, however, disordered eating behaviours can become so ingrained that they will develop into a diagnosable eating disorder among both female and male athletes.^2,3^ Eating disorder diagnosis in athletes is beyond the scope of this research; however, it aims to highlight how disordered eating behaviours exist among South African amateur athletes. Athletes are often under pressure to perform at best because of the nature of competition and it is not clear whether amateur athletes are engaging in similar disordered eating behaviour than some professional athletes that put them at risk for developing an eating disorder. By identifying the disordered eating behaviours of athletes, it is possible to identify the ‘at risk’ amateur athlete with the potential development of eating pathology.

The idea of an ‘at risk’ of an eating disorder athlete is underestimated and needs more attention especially when viewed in light of the prevalence of eating disorders in sport.^4^ Research focussing on the prevalence of eating disorders among different athletes is largely inconclusive because of a number of mitigating factors, but it seems to be more prevalent among athlete than non-athlete populations,^5^ both during adolescence^4^ and adulthood.^6^ Prevalence rates also seem to be higher among female athletes than male athletes.^4,6^ Weight-sensitive sports such as endurance, weight category and aesthetic sports, jumping sports and dancing^5^ seem to be high-risk sports for the development of eating disorders or inappropriate behaviours when engaging with food.^6^ International studies have reported high variability in the prevalence rates of athletes at risk for eating disorders, from as low as 7% to as high as 62%.^4,6,7^ It is argued that the variability may be because of diagnostic and methodological issues.^1^ Similar inconsistent prevalence rates were found among South African athletes, from as low as 14% among runners^8^ to a substantially higher number of 54% among elite netball players^9^ and 62.5% in student track and field athletes.^10^ Therefore, research on eating disorders in sport needs to be understood and interpreted with caution as results are often inconsistent. This necessitates more research to understand the disordered eating behaviours and its prevalence in amateur sports in South Africa. Similar research focussing on identifying the amateur athlete ‘at risk’ of developing disordered eating behaviours needs to be more prominent in order to assist in the prevention of the development of any eating pathology, including the role competitive anxiety^11^ plays in the context of amateur club-level sport.

## Method

Previous international research^1,2,4,5,6,7^ focussing on eating disorders among sportsmen and sportswomen was conducted mostly on professional youth, student and adult athletes, often overlooking disordered eating behaviours among amateur athletes at club level. This study is exploratory in nature and attempts to determine the prevalence and risk factors of disordered eating behaviours and to identify amateur athletes ‘at risk’ of developing eating disorders at club level in South Africa. Group differences were determined for gender, type of sports and level of participation. The study also explored the relationship between variables and predictability of disordered eating behaviours among amateur athletes at club level in South Africa.

## Participants

A purposive sample of 278 athletes with a mean age of 27 ± 11.30 years from various amateur sports clubs representing 31 team and individual sports codes in urban areas in and around Johannesburg, Gauteng, were invited to participate in the study through a study information letter. Clubs that responded positively to the invitation were included in the study. The sample included both male and female athletes, who participated in provincial, national and international levels. Twenty field workers met with the athletes at their sport clubs and introduced about the purpose of the study. Athletes who volunteered to participate were asked to sign an informed consent form that ensured confidentiality, defined the parameters of the study and how the data will be used and published. Once the informed consent was signed, participants completed the questionnaires.

## Research instrument

Athletes were asked to complete a biographical questionnaire, including their age, gender, weight, height, type of sports and level of participation. They were also requested to complete the Eating Attitudes Test-26 (EAT-26)^12^ and the Sport Competition Anxiety Test (SCAT).^11^

*Eating Attitudes Test-26* is a widely used standardised measure of concerning symptoms that are characteristic of eating disorders.^12^ The EAT-26 does not provide a definitive diagnosis of a specific eating disorder, but rather serves as a screening measure to assess or identify individuals exhibiting symptoms of eating pathology that needs attention.^12^ It consists of 26 questions answered on a six-point Likert scale, ranging from ‘always’ to ‘never’. Three subscales, dieting, bulimia and food preoccupation as well as oral control can be calculated from the 26 questions. The subscales can be defined as follows:^12^

1. Dieting is an indication of a pathological avoidance of fattening (high-calorie) foods and food preoccupation. Sport athletes with high scores on this scale are usually dissatisfied with their body shape and have a desire to be smaller.
2. Bulimia and food preoccupation is an indication of body image disturbance positively related to bulimia (binge eating and purging behaviour) and associated with a heavier body weight.
3. Oral control refers to self-control regarding food and social pressures to lose weight. The questionnaire also includes a section on behavioural assessment (four questions) to determine excessive weight control measures.

A score of 20 and above denotes a need to be referred to a professional to assess the presence of an eating disorder. High scores are not necessarily indicative of an eating disorder, but suggest concerns about an athlete’s weight, shape and eating behaviours. Similarly, low scores are not necessarily indicative of the absence of such concerns, but should be read with caution because of denial of symptoms, which could occur in self-report instruments.^12^ The EAT-26 has high validity and reliability, with Cronbach’s alpha scores between 0.83 and 0.90 for the three subscales and 0.90 for the scale as a whole.^12^ It must be noted however that the EAT-26 is a screening instrument with low predictive power^13^ and therefore results have to be interpreted with caution. High scores on the instrument must not be interpreted as an eating disorder without further screening or referral to a professional, as questions can be misinterpreted, specifically in cross-cultural contexts such as South Africa.

The SCAT^11^ consists of 15 questions of which 10 questions are used to calculate a competitive anxiety score. It is answered on a three-point Likert scale ranging from ‘hardly ever’ to ‘sometimes’ and ‘often’. Norm scores were developed by the author^11^ and can be categorised to identify athletes with low (anxiety score < 16), moderate (anxiety score = 17–22) and high competitive anxiety (anxiety score > 23). High reliability and validity is reported on the instrument with an internal consistency (Cronbach’s alpha) of between 0.8 and 0.85.^11^

## Data analysis

Descriptive statistics (means and standard deviations) and frequency tables were used to describe the participants. Statistical significance was set at 5% (*p* = 0.05). The prevalence of disordered eating and risk factors was calculated as a percentage according to the norm scores provided with the EAT-26^12^ and the internal consistency of the various subscales was determined with Cronbach’s alpha. Group differences were determined using independent samples *t*-tests and analysis of variance (ANOVA) with Bonferonni post hoc tests. Finally, to determine which factors have a statistically significant contribution to the prediction of disordered eating, a logistic regression was performed.

## Ethical consideration

This research project was approved by the University of Johannesburg’s higher degrees and ethics committees before being conducted.

## Results

The amateur athletes who participated in this study spent on average 8.71 ± 5.79 h training per week. While most of them participated in club levels (*n* = 178, 64.7%), the sample included amateur athletes participating in provincial (*n* = 37, 13.5%) and national or international (*n* = 60, 21.8%) levels. The sample consisted of 161 (57.9%) male and 117 (42.1%) female participants, representing a fair distribution to determine group differences. Athletes from various team sports (*n* = 132, 47.5%) and individual sports (*n* = 146, 52.5%) were represented in the sample (see Table 1). The team sports were mostly represented by Football (37.1%), Rugby (16.7%), Basketball (14.4%) and Netball and Hockey (7.6%). Athletics (36.3%), Tennis and Boxing (9.6%) and Squash (7.5%) were represented mostly in individual sports.

**TABLE 1:** Frequencies of team and individual sports represented in the sample.

Sport	Frequency (*n*)	%
**Team sports**		
Football	49	37.1
Rugby	22	16.7
Basketball	19	14.4
Netball	10	7.6
Hockey	10	7.6
Cricket	5	3.8
Dancing	5	3.8
Sailing	4	3.0
Water Polo	3	2.3
Touch Rugby	2	1.5
Drum Majorette	2	1.5
Softball	1	0.8
**Total**	**132**	**47.5**
**Individual sports**		
Athletics	53	36.3
Tennis	14	9.6
Boxing	14	9.6
Squash	11	7.5
Gymnastics	7	4.8
Judo	7	4.8
Swimming	6	4.1
Cycling	6	4.1
Karate	5	3.4
Golf	4	2.7
Aerobics	4	2.7
Wrestling	3	2.1
Canoeing	3	2.1
Ballet	2	1.4
Weight lifting	2	1.4
Kickboxing	2	1.4
Rowing	1	0.7
Lawn bowls	1	0.7
Yoga	1	0.7
**Total**	**146**	**52.5**

The physical attributes and eating attitudes of the participating amateur athletes are presented in Table 2. The participants had a mean weight (kg) of 68.87 ± 14.20 ranging between a minimum of 43 kg and a maximum of 118 kg. The mean height (m) of the participants was 1.69 ± 0.14, with a minimum of 1.20 m and a maximum of 2.30 m. These measures were used to calculate the body mass index (BMI) of the participants. The mean BMI (24.33 ± 4.75) of the participants is within the normal category (presented in Table 2); however, upon further analysis, 5% of the participants were found to be underweight, while 23.6% and 11.6% were found to be categorised as overweight and obese, respectively.

**TABLE 2:** Physical attributes, competitive anxiety (Sport Competition Anxiety Test scores) and eating attitudes (Eating Attitudes Test-26scores) of the sample.

Variable	Mean ± s.d.	Minimum	Maximum	Categories	Frequency	
					*n*	%
Body mass index	24.33 ± 4.75	12.70	45.14	Underweight	13	5.0
				Normal	155	59.8
				Overweight	61	23.6
				Obese	30	11.6
**SCAT scores**						
Competitive anxiety	20.45 ± 4.13	10	30	Low	35	12.6
				Moderate	151	54.3
				High	92	33.1
**EAT-26 scores**						
Eating attitude	10.59 ± 9.55	0	55	No risk – ED	233	85.3
				Risk – ED	40	14.7
Dieting	8.49 ± 6.02	2	31	Risk – AN	20	7.30
Bulimia and food preoccupation	2.14 ± 2.44	0	12	Risk – BN	6	2.19
Oral control	2.32 ± 2.68	0	13	High OC	14	5.11

ED, eating disorder; AN, anorexia nervosa; BN, bulimia; OC, oral control; SCAT, Sport Competition Anxiety Test; EAT-26, Eating Attitudes Test-26; s.d., standard deviation.

**TABLE 3:** Disordered eating behaviour.

Behaviour	Indicator	Frequency	%
Binge eating	No	218	79.6
	Yes	56	20.4
Vomiting to control weight	No	240	87.6
	Yes	34	12.4
Laxatives to control weight	No	214	78.1
	Yes	60	21.9
Exercise to control weight	No	258	94.2
	Yes	16	5.8
Lost 9 kg (20 lb) or more in the past 6 months	No	231	84.3
	Yes	43	15.7

Both the SCAT and EAT-26 instruments yielded high reliability scores, with Cronbach’s alpha at 0.829 and 0.841, respectively, and thus contributed much towards the internal validity of the study. The results of the study could therefore be reported with confidence. The athletes’ mean SCAT score for competitive anxiety (20.45 ± 4.13) fell within the moderate parameters of the test, which is appropriate. However, upon further analysis, one-third (33.1%) of the athletes in the study seemed to have high competitive anxiety (see Table 2).

Table 2 also presents the EAT-26 scores of the participants. According to the norm scores,^12^ a score of 20 and more might be indicative of an athlete at risk of developing an eating disorder (subclinical eating disorder pathology). Although the mean score (10.59 ± 9.55) for these athletes is well below the cut-off score of 20, further analysis and categorisation of the scores showed that 14.7% of the amateur athletes in this sample seem to be at risk of developing an eating disorder.

The disordered eating behaviour of the athletes (Table 3) provides an indication of the pathological attempts of amateur athletes to lose weight rapidly. Although most of the participants did not engage in such behaviours, 21.9% used laxatives, 20.4% engaged in binge eating and 12.4% purged to lose weight. In contrast, only 5.8% used exercise to lose weight. A total of 15.7% of the participants lost more than 9 kg (20 lb) in the past 6 months.

Gender, sport, level of participation and anxiety group differences for disordered eating are presented in Table 4. Although no group differences were found for the type of sports, the level of sport participation and the competitive anxiety of the athletes, a significant difference for disordered eating was found for the gender groups, with female athletes having significantly higher disordered eating scores (*M* = 12.91 ± 10.53; *t* [272] = -3.53, *p* = 0.000) than male athletes (*M* = 8.87 ± 8.38) in the study. This implies that female athletes were much more ‘at risk’ to engage in problematic eating behaviours, putting them at greater risk of developing an eating disorder. The difference between the means yielded a small effect size (eta-squared = 0.044). A chi-square test for independence indicated a significant association between gender and risk for an eating disorder (*c*^2^ [1, *n* = 273] = 6.75, *p* = 0.009, phi = 0.168), with only 9.6% of male athletes but 21.6% of female athletes at risk for developing an eating disorder.

**TABLE 4:** Group differences for disordered eating among amateur athletes.

Variable	Group	Mean ± s.d.	Sign. (*p*)
Gender	Male	8.87 ± 8.38	0.000
	Female	12.91 ± 10.53	
Type of sports	Team sports	10.30 ± 10.20	0.639
	Individual sports	10.85 ± 8.98	
Level of participation	Club	9.83 ± 9.10	0.275
	Provincial	12.14 ± 9.21	
	National or international	11.42 ± 10.21	
Competitive anxiety	Low	8.47 ± 7.54	0.282
	Moderate	10.52 ± 9.78	
	High	11.52 ± 9.80	

s.d., standard deviation.

A logistic regression analysis was performed to determine which factors play a significant role in the likelihood of amateur athletes developing an eating disorder. The logistic regression model consisted of nine independent variables (see Table 5): gender, type of sports, level of participation, BMI, anxiety, binge eating, vomiting to control weight, use of laxatives to lose weight and exercise to control weight. The model was statistically significant with the omnibus test of model coefficients (*χ*^2^ [9, *n* = 254] = 25.32, *p* = 0.003), while the Hosmer-Lemeshow goodness-of-fit test was not statistically significant (*χ*^2^ [8, *n* = 254] = 9.27, *p* = 0.320). Thus, both tests indicated that the model was able to distinguish between participants who were at risk and who were not at risk of developing an eating disorder.

**TABLE 5:** Variables in the equation of the logistic regression.

Variables	*B*	SE	Wald	*df*	Sig.	Exp(*B*)	95% CI for Exp(*B*)	
							Lower	Upper
Gender	1.158	0.426	7.378	1	0.007	3.183	1.380	7.339
BMI	0.084	0.037	4.994	1	0.025	1.087	1.010	1.170
Anxiety	−0.029	0.050	0.340	1	0.560	0.971	0.880	1.071
Type of sports	−0.164	0.400	0.168	1	0.682	0.849	0.388	1.860
Level of participation	0.948	0.402	5.548	1	0.019	2.581	1.173	5.680
Binge eating	0.879	0.454	3.748	1	0.053	2.407	0.989	5.859
Vomiting	0.771	0.570	1.830	1	0.176	2.162	0.707	6.607
Laxatives	0.018	0.484	0.001	1	0.971	1.018	0.394	2.629
Exercise	0.066	0.741	0.008	1	0.929	1.069	0.250	4.563
Constant	−4.476	1.494	8.971	1	0.003	0.011	-	-

Note: Variable(s) entered on step 1: All.

BMI, body mass index; SE, standard error; CI, confidence interval.

The model explained between 9.50% (Cox and Snell’s *R*^2^) and 16.6% (Nagelkerke’s *R*^2^) of the variance in the risk to develop an eating disorder and classified 85% of the cases correctly. As presented in Table 5, three of the factors – gender (*p* = 0.007), level of participation (*p* = 0.019) and BMI (*p* = 0.025) – made a statistically significant contribution to the model. The strongest predictors of risk to develop an eating disorder were gender (odds ratio: 3.18), level of participation (odds ratio: 2.58), binge eating (odds ratio: 2.41) and vomiting to control weight (odds ratio: 2.16). This indicates that amateur athletes who are female, participate at provincial or international level, binge eat and purge to lose weight are two to three times more likely to be at risk of developing an eating disorder, when controlling for all the other factors in the model.

## Discussion

The results presented above reflect those of a purposive sample of amateur athletes from various sports clubs in Johannesburg, Gauteng, and as such cannot be generalised to other contexts. The results, however, indicate that there could be concerns for athletes being at risk of developing eating disorders, as disordered eating behaviour is prevalent in this group of athletes. The prevalence rate of 14.7% for amateur athletes at risk of developing an eating disorder participating at club level is consistent with the results found in other international studies.^4,6,7^ However, the results of those studies determined the prevalence rate of professional athletes. This suggests that the risk of developing an eating disorder is just as prevalent among club sport athletes as for professional sport athletes. Although the type of sport, the level of participation and anxiety did not indicate differences in the risk to develop an eating disorder, the results indicated that female athletes are more at risk of engaging in disordered eating behaviours, leaving them vulnerable to eating disorders. This result is consistent with previously conducted international studies in sport.^1,2,3,14^ This is an indication that female athletes at amateur level are just as vulnerable to eating disorders as their international counterparts, and sports managers and coaches should thus be aware and sensitive not to aggravate the issue and, as a preventative strategy, should refer athletes to psychiatrists or clinical psychologists when disordered eating behaviour is observed. Female athletes are perhaps more prone to the development of eating disorders because of body dissatisfaction and as a result pre-occupied with food, body weight and appearance.^14^ The results of this study show that gender, level of participation and weight control strategies are important indicators associated with the risk of developing an eating disorder. Factors influencing this need could arise from social media exerting the ideal symbolisation of health and fitness, and female sport athletes comparing themselves to their fellow sport athletes. However, female athletes reported that they are more likely than their male counterparts to use exercise to control their weight, mood, health and body tone.^15^

Furthermore, the results suggest that for the most part, the eating attitudes of the amateur athletes who participated in the study were within normal limits. However, more than one in five athletes indicated that they engaged in binge eating behaviour along with using laxatives to control weight. Amateur athletes in this study were less likely to use exercise as a means to lose weight and would rather engage in purging or using laxatives to do so. This could be attributed to gender, as women generally suffer from disturbed eating behaviours.^16^ The results of the study also indicated that there is a significant gender difference regarding eating disorder risk, thus making it more likely that female athletes would express that they have engaged in binge eating and the use of laxatives to control weight.^17^ Researchers have found that female athletes are more likely to report and engage in weight loss behaviours or disordered eating such as binge eating, rigorous dieting, fasting, vomiting and the use of laxatives and diuretics compared to men.^16,17^

It must be noted that weight loss among athletes is not necessarily related to an eating disorder. However, one can assume from the results of the study that a fair amount of amateur athletes who engage in at least one of the weight control measures have at least one risky eating behaviour. This could possibly leave them vulnerable to a perpetual cycle of not knowing the correct nutritional information needed, thus leading to disordered eating behaviours. These research findings coincide with other research,^18^ reporting that a pattern of eating habits of dieting during an athletic career may predispose athletes to later and more severe eating disorders within both genders. It seems that gender, level of participation and BMI of the athletes are important contributors to the prediction of the risk of developing an eating disorder. A model that included nine factors and was able to correctly classify more than 85% of cases can be utilised by coaches and medical or health professionals involved to be more aware of athletes who are at risk of an eating disorder. Officials, such as coaches who are aware of athletes who are overweight and engaged in disordered eating behaviour, should consult medical or health professionals available to the athletes to address the risk sensitively and appropriately.

Limitations of the study are primarily methodological in nature. The sample was purposive and only selected from a local population in Johannesburg. The results are thus not generalisable. Only athletes from clubs who responded to the researchers’ invitation were included; this could contribute to the possible underestimation of the results because of non-participation. It is suggested that future research strategies should include random selection of participants. Furthermore, although the sample included a variety of 31 different sports codes, it is relatively small and could be expanded in future research to include a larger population of athletes representative of other provinces in South Africa, as well as athletes from rural populations. The sample is also non-clinical and heterogeneous as the purpose of the study was exploratory. Future research should be conducted to include clinical homogeneous populations in sports that could be more advantageous to psychiatry. Another limitation of this study is that no pilot study was conducted to attend to possible problems with item interpretation. Although the SCAT was validated for the South African context,^19^ it is not so for the EAT-26, as its predictive power is low and results would need to be confirmed by further screening by professionals. Although both questionnaires had high internal consistency, it is suggested that future research should be conducted on validating the EAT-26 for the South African context.

## Conclusion

Indicators of eating disorder risk among club-level athletes are gender, BMI, level of participation, binge eating, vomiting and using laxatives to control weight. These behaviours predominately found in female amateur athletes seem to put them at a greater risk of developing an eating disorder. All of the disturbed eating patterns that female amateur sport athletes seem to engage in are consistent with people who may have subclinical symptoms of anorexia nervosa, bulimia nervosa, binge eating disorder or eating disorders not otherwise specified.
